# Indiana State Dental Society

**Published:** 1868-08

**Authors:** 


### INDIANA STATE DENTAL SOCIETY.

This Society met in Indianapolis, according to announcement, June 30, and continned in session three days. We had the pleasure of spending two days at the meeting; and although suffering from too great nervous prostration to enjoy things enjoyable as well as formerly, we found it refreshing and "good to be there."

The meeting was well attended, though there are scores of Dentists in the State who did not attend, who can give no valid excuse for their absence.

The discussions, in spite of the "heated term," were mostly practical and lively. Indeed, the Society appeared like a band of brothers, met for mutual consultation in reference to common interests. No one appeared to have an "axe to grind;" but each seemed willing to take a turn at the crank, that whatever needed sharpening might be ground. Our last visit to this Society was with the mercury at zero. This time it was rather higher—about to two zeroes with a unit preceding. Both meetings were lively and profitable, proving that *heads* and *hearts*—not the weather—are the special agencies in professional progress.

The subject of anæsthesia was considered at some length. We had the opportunity—and gladly embraced it—to set forth more fully than previously, the results of a long, complex series of experiments and researches on NITROUS OXYD, for which—the difficult and dangerous experiments, not the talk, of course—we received from the Society the most substantial vote of thanks ever awarded us; and we must say, that the quiet, cordial, unostentatious manner in which the affair was conducted, more than doubles our estimation of it. It appears, for we were out at the time, that the vote was by ballot. We saw but *two* tickets. They were *green* on the back; and, probably, on account of their peculiar markings, we could realize a hundred dollars for them any day.

If we knew what more to say about it, we'd say it. We think over the members of the Indiana State Society, one at a time, (including Messrs. Strong and Smith) and ask that our Father in Heaven may be their Father in Heaven, that we may be joint heirs of "an inheritance incorruptible, and undefiled, and that fadeth not away." W.

